# Supplementary material for: Are carbon emissions trading and green financial instruments synergistic? -Comprehensive quantitative research based on content analysis
Source: PLoS One. 2024 Mar 7;19(3):e0298601. doi: 10.1371/journal.pone.0298601 (PMC10919601; doi:10.1371/journal.pone.0298601)
Supplement: S1 Data — (ZIP) [file pone.0298601.s001.zip › Data/地级市Z_F工作报告.zip!/╕ú╜¿/─■╡┬╩╨/└┤╫╘╣1⁄2╓┌║┼í╛┬φ┐╦╩2╛▌═°í┐_2013.docx]

各位代表：

现在，我代表宁德市人民政府，向大会报告政府工作，请予审议，并请市政协委员和其他列席的同志提出意见。

一、2012年政府工作回顾

2012年，在省委、省政府和市委的坚强领导下，我市各级政府深入贯彻落实科学发展观，按照打造福建科学发展加快发展新增长极要求，以解放思想为先导，以“五个年”活动为抓手，全面实施环三发展战略，凝心聚力，锐意进取，顺利实现“千百万”目标，较好地完成了市三届人大一次会议确定的各项任务。

（一）攻坚克难积极作为，经济增长稳中有进

积极主动应对宏观经济环境持续紧缩的压力，坚持把稳增长作为首要任务，加强对重点行业、重点企业和新增长点的跟踪服务，出台扶持电机电器千亿产业集群、发展总部经济、支持品牌建设、帮扶小微企业、促进改制上市等一系列政策措施，推动实体经济持续健康发展。多措并举突破资金瓶颈制约，积极发挥金融机构主渠道作用，率先在全省发行中小企业区域集优票据，各类金融机构年末融资余额1605亿元、增长13%；积极向上争取支持，全年争取各类转移支付资金83.66亿元，比上年增加11.82亿元；新组建市交通、港务、城建、旅游、能源五大集团，强化国有资本运作和投融资体制创新，筹集建设资金31.3亿元。初步统计，全市完成地区生产总值1075亿元，跨过千亿目标，增长13%；财政总收入104.4亿元，跨过百亿目标，增长25.2%，公共财政预算收入70.6亿元、增长30.1%；城镇居民人均可支配收入21880元，跨过2万元目标，增长13.3%；农民人均纯收入9075元，向万元目标迈进，增长17%，经济实力迈上新的台阶。

（二）调整结构促进转变，产业提升步伐加快

农业基础不断巩固。落实强农惠农富农政策，全市农林牧渔业总产值362亿元、增长5.8%。农民专业合作社加快发展，农业产业化水平不断提升，新增市级龙头企业34家。解决32.1万农村居民饮水安全问题，开工建设重大水利项目14个，新建标准化渔港10个，除险加固水库43座、海堤11条，新建和改造农村公路410公里，“村村通客车”率达93%。完成836个村庄规划编制。实施旧村复垦项目174个，建设高标准基本农田3.05万亩，补充耕地1.45万亩。防灾减灾工作扎实有效，农机、气象、水文、防震等服务保障有力。

工业经济优化提升。坚持抓龙头、铸链条、建集群，加快推进新型工业化，全市规模以上工业总产值1740亿元、增加值445亿元，分别增长17.5%和19.8%，增幅连续八年位居全省首位。加快传统产业转型升级，完成技改投资65亿元、增长110%。新认定省级战略性新兴产业企业53家，新增中国驰名商标11件，32家企业列入省重点上市后备企业，广生堂药业、三祥新材上市材料已上报中国证监会审核。园区建设扎实推进，新增园区面积4.2万亩，东侨经济开发区升格为国家级经济技术开发区。

第三产业稳步发展。新增限额以上商贸流通企业93家，新建和改造集贸市场6个、市级副食品基地52个，全社会消费品零售总额318亿元、增长16.6%；物价保持稳定，居民消费价格总水平涨幅控制在2.6%。城市商业业态有效提升，宁德万达广场投入运营，声立方呼叫中心、红星美凯龙家居广场、翠屏路步行街等一批城市综合体开工建设。成功举办第二届宁德世界地质公园文化旅游节，白水洋·鸳鸯溪被评为5A级景区，全市接待游客超过1000万人次、旅游收入77亿元，分别增长20%和22%。金融服务业加快发展，分别与兴业银行以及建设银行、中国银行福建省分行签订战略合作协议，交通银行入驻我市，中信银行宁德分行、福安渝农商村镇银行获批筹建。

（三）比学赶超乘势而上，项目带动成效显著

“五大战役”深入推进。建立健全领导挂钩、首席责任人、“三争三比”等项目工作机制，促进“五大战役”深入实施，完成投资766亿元，超年度计划26%。福建首台核电机组——宁德核电站1号机组并网发电，鼎信30万吨镍合金建成投产，宁武高速公路宁德段提前半年通车；联德镍合金、宁德新能源科技二期等一批产业项目，沈海复线柘荣至福安段、福寿高速公路等一批基础设施项目，市图书馆、妇女儿童活动中心等一批民生项目开工建设；大型钢铁项目选址环境可研报告通过评审，衢宁铁路纳入国家“十二五”规划并启动前期工作。

“三维”对接成果丰硕。不断提升与央企、民企、外企的合作层次和效益，有效深化与东南亚、港澳台以及长三角、珠三角、闽南等地的区域合作，着力引进一批龙头项目、配套项目和关联项目，全年共签约“三维”项目444项，总投资2214亿元，当年签约列入跟踪管理的141个项目已开工80个，开工率56.7%。

（四）多措并举建管并重，城乡面貌日益改善

中心城市建管加强。新一轮城市总体规划获批实施。市政基础设施建设扎实推进，基本完成城区5条主干道沥青路面改造，铺设雨污分流管道10.2公里，市第三水厂、贵岐山污水处理厂二期、北区污水处理厂建成运营，金溪防洪堤加快建设，5个城区低洼地防洪排涝工程全面启动。城市管理不断强化，环卫园林管理体制进一步理顺，“清违”工作持续开展，拆除“两违”建筑面积3.65万平方米，城市环境卫生、河流湖泊、建筑渣土、户外广告牌整治取得阶段性成效。城市建成区绿化覆盖率达40.5%，人均公共绿地面积13.8平方米，顺利通过国家级园林城市省级初检。

城乡发展步伐加快。县域经济实力不断壮大，产业基础进一步夯实。福鼎、福安蝉联全省县域经济发展“十佳”。小城镇改革发展“4+15”试点建设扎实推进，产业、人口等要素加快集聚，城镇化率达50.5%。第二批新农村示范村加快建设，第三批整村推进扶贫开发有序开展，完成“造福工程”搬迁4985户20902人。出台帮扶民族乡政策，集中资源支持民族乡村加快发展。

生态质量持续提升。推进“点线面”综合整治，植树造林41.9万亩，沈海高速森林生态景观通道建设全面完成，综合治理水土流失面积18.75万亩，清理海上无序养殖5200多亩。创建国家级生态乡镇21个、省级生态乡镇32个、市级以上生态村838个、“绿色村庄”160个，537个村通过省级“家园清洁行动”验收，完成农村环境连片整治项目2个。落实环保“一岗双责”，大力推进节能减排，主要污染物排放得到有效控制，万元GDP能耗进一步下降。境内主要流域水环境功能区和饮用水源水质达标率均达100%，生态环境质量继续保持优良水平。

（五）深化改革扩大开放，发展动力有效增强

林权制度配套改革深入推进，森林综合保险面积1050万亩；建制村集体土地所有权发证基本完成。行政审批制度改革取得新成效，市级审批事项进一步精简39%。国有企业公司制、股份制改革步伐加快，新改制市属企业5家。外经外贸稳步提升，全年验资口径实际利用外资1.2亿美元、增长26.2%；出口总额20.5亿美元、增长42%。宁台交流合作进一步深化，成功举办第三届海峡两岸电博会、第四届海峡论坛·陈靖姑文化节和第四届海峡摄影展。

（六）以人为本增进和谐，社会建设协调推进

民生保障不断改善。进一步加大公共财政对民生的投入，全市各级财政用于民生支出66.43亿元、增长23%，36件为民办实事项目全部完成。新增城镇就业2.58万人、转移农村劳动力4.18万人，城镇登记失业率控制在2.04%。新开工保障性住房8936套，竣工和基本建成6888套，历年配租配售率达86.28%。全民医保基本建立，城乡居民社会养老保险实现制度全覆盖。城乡低保实现应保尽保，农村低保年标准从1200元提高到1800元。社会救助体系进一步健全，建成县级社会福利中心3个、社区综合服务站12个和各类养老机构201个。

社会事业加快发展。争取国家和省级科技项目120项，比上年增加50项，2个项目列入科技部“863”计划，闽东中小电机产业集群列入国家创新基金扶持项目，福安被认定为国家火炬中小电机特色产业基地。新改扩建公办幼儿园39所，完成城区中小学扩容12所，提前一年全面实现“双高普九”目标，东侨经济开发区通过义务教育基本均衡省级验收，宁德师范学院新校区一期、二期工程全面完成。医药卫生体制改革稳步推进，新增医疗机构床位1123张，村级卫生所推行基本药物零差率销售，县乡村三级医疗体系进一步完善。人口与计生工作取得新成绩，低生育水平进一步得到巩固，人口自然增长率6.6‰，柘荣被评为“全国计划生育优质服务县”。全民健身活动广泛开展，建成市老年人体育活动中心，完成640个建制村农民体育健身工程建设。

文化建设扎实推进。文化体制三项改革稳步实施。“魅力闽东”文化惠民演出常态开展，成功举办福建省首届“三月三”畲族文化节，电影《爱在廊桥》、《为你而来》分获国家“五个一工程”优秀作品奖和贡献奖。一批县级文化体育场馆相继建成，新建和改造激情广场示范点20个、农家书屋470家，20户以上自然村全面实现广电“村村通”。积极扶持文化产业加快发展，推出首批13条文化旅游精品线路，宁德（霞浦）国际滨海影视文化创意产业园、宁德工艺博览城等重大文化产业项目加快推进。

社会管理有效加强。“六五”普法深入开展，“平安宁德”建设持续深化，公安综合警务机制改革稳步推进，全面构筑打防控一体化体系，群众对社会治安满意率达95.65%。积极创新社会管理方式方法，总结推广“霞浦经验”，深入排查调处各类矛盾纠纷和不稳定因素，按“路线图”处理群众信访答复率达100%。食品药品安全监管不断强化。安全生产“一岗双责”有效落实，“打非治违”专项整治行动深入开展，道路交通安全综合整治“三年行动”和大会战取得阶段性成效，全市安全生产形势保持稳定。

（七）依法行政优化服务，自身建设不断加强

自觉接受人大及其常委会监督，依法执行人大决定决议，认真办理人大代表建议、批评和意见，积极配合人大常委会开展执法检查、人大代表视察及专项调研、专题询问。支持人民政协履行政治协商、民主监督、参政议政职能，认真办理政协提案，积极采纳委员调研成果。广泛听取各民主党派、工商联、各人民团体、无党派人士及社会各界人士的意见。全年共办理市人大代表建议267件、市政协提案291件，办结率均为100%。进一步完善行政复议工作，加强行政执法监督，强化政府信息公开。加强政府领导班子思想政治作风建设，落实“无会周”制度，深入开展“四下基层、四解四促”活动，积极帮助企业、基层协调解决具体困难和问题。机关效能建设继续深化，行政监察和审计监督不断加强，廉政建设和反腐败工作扎实推进，政府公信力、执行力稳步提升，全市上下进一步形成了风正气顺、人和业兴的良好氛围。

此外，人事、编制、统计、审计、档案、老区、库区、外事侨务、宗教、老龄、老干部、地方志、残疾人、关心下一代、国防动员、人防、民兵预备役、双拥支前、海防、打私等工作都取得新成效。

成绩的取得来之不易，这是省委、省政府正确领导和关心支持的结果，是在市委坚强领导下全市人民团结一致、艰苦奋斗的结果，是市人大、市政协和社会各界加强监督、鼎力支持的结果。在此，我代表市人民政府，向为宁德发展付出辛勤劳动的全市人民，向给予政府工作有力支持和有效监督的人大代表、政协委员、各民主党派、工商联、各人民团体、无党派人士、离退休老同志和社会各界人士，向为宁德发展作出积极贡献的中央及省属驻宁机构、驻宁部队、武警官兵、公安干警、企业界人士，向关心支持宁德发展的港澳同胞、台湾同胞、海外侨胞、国际友人，表示衷心的感谢和崇高的敬意！

在看到成绩的同时，我们也清醒认识到经济社会发展和政府工作还存在不少的困难和问题，主要是：经济总量较小，发展基础仍显薄弱，受国内外经济大环境影响，地区生产总值未能完成增长15%的年度预期目标；科技创新能力不足，产业转型升级任务艰巨；要素保障不够有力，项目落地难问题比较突出；中心城市辐射带动能力不强，县域经济发展不平衡，城乡规划建设和管理水平亟待提升；民生保障能力水平与人民群众期盼还有较大差距，社会管理面临一系列的新问题新挑战；机关作风有待进一步改进，干部担责和开拓意识不强，行政不作为和消极腐败现象在个别单位和领域仍然存在。我们要高度重视这些问题，采取更加有力的措施切实加以解决。

二、2013年政府工作主要任务

2013年是全面贯彻落实党的十八大精神的第一年，是实施“十二五”规划承前启后的关键一年，也是“六新大宁德”建设的开局之年。政府工作的总体要求是：全面贯彻党的十八大、中央经济工作会议和省委九届六次全体（扩大）会议精神，以邓小平理论、“三个代表”重要思想、科学发展观为指导，紧紧围绕提前三年与全省同步全面建成小康社会的目标，认真落实市委关于建设“六新大宁德”、打造福建科学发展加快发展的新增长极、跻身全省“第二方阵”的决策部署，全力实施环三发展战略，更加注重稳中求进、好中求快，更加注重产城并重、城乡一体，更加注重以人为本、民生优先，加快建设更加优美更加和谐更加幸福的宁德。

经济社会发展的主要预期目标是：地区生产总值增长13%；财政总收入增长15%；全社会固定资产投资增长25%；社会消费品零售总额增长16%，居民消费价格总水平涨幅控制在4%以内；出口总值增长15%，验资口径实际利用外资增长20%；城镇居民人均可支配收入增长15%，农民人均纯收入增长17.5%；城镇登记失业率控制在4%以内；人口自然增长率控制在7.3‰以内；节能减排指标控制在省定目标以内。

这些预期目标，综合考虑了国内外经济环境变化的影响，与我市“十二五”规划以及全面建成小康社会目标相衔接，体现了速度与质量的协调、富民与强市的统一。我们要紧紧抓住党的十八大带来的历史机遇和重要条件，贯彻落实市委提出的构建高质高效新产业、宜业宜居新城镇、畅通便捷新交通、丰富多彩新文化、幸福和谐新民生、优质优美新环境的“六新大宁德”的决策部署，以科学规划为引领，以质量效益为中心，以项目带动为抓手，“软件”和“硬件”并举，当前和长远结合，确保经济持续健康发展和社会和谐稳定，实现“六新大宁德”建设开好局、起好步。重点抓好以下几个方面工作：

（一）夯实“三农”基础，加快发展现代农业

增强农业综合生产能力。稳定粮播面积，提高粮食单产，抓好粮食储备，确保粮食安全。实施现代农业示范工程，推进省级农业标准化示范基地、农民创业园和11个千亩以上高优农业示范园区建设，培育10家成长型特色农产品精深加工示范企业，规范和扶持发展150家省、市级示范专业合作社，构建集约化、专业化、组织化、社会化相结合的新型农业经营体系。加大农业“五新”推广力度，提升茶叶、水产、食用菌三大主导产业，促进由产量大市向产业强市转变；壮大林竹、畜牧、果蔬、中药材等优势特色产业；培育生物质能源、生物种业等新兴产业；扶持发展精致农业、观光农业、生态农业；新增一批农产品地理标志证明商标，促进农业产业往高端化、品牌化发展。加强绿色生产，从源头确保农产品质量安全。推进防灾减灾体系建设，加强防汛抗旱、动植物疫病防控和森林资源保护。

完善农业农村基础设施。续建新建官昌水库、赛江防洪工程二期等一批水利项目，加快上白石水利枢纽工程、吴坑水库、田螺岗水库以及东冲半岛人饮工程等项目前期工作。高质量实施蕉城、屏南、寿宁、周宁等小型农田水利重点县（区）建设，除险加固一批海堤、水库、水闸工程，建成中小河流治理项目15个，新增节水灌溉面积1.6万亩，解决30万农村居民饮水安全问题。建成霞浦三沙中心渔港，加快建设福鼎沙埕中心渔港，新建续建25个标准化渔港。

落实强农惠农富农政策。继续加大对“老、少、边、岛”地区和库区移民帮扶力度，积极推进产业扶贫，深化省级扶贫开发重点县、市级扶贫开发重点乡镇帮扶共建成果，完成第三批整村推进扶贫开发任务。扶持新建一批50户以上“造福工程”集中安置区，完成搬迁3万人。着力缩小农民收入与全省平均水平的差距，鼓励和指导农民优化种养结构，延伸发展家庭手工业、农村服务业，增加经营性收入；启动农村产权“五证”发放工作，鼓励农民把资产转化成资本，增加财产性收入；提高农村劳动力转移“阳光工程”培训针对性，推动贫困农户“一户一培训、一户一就业”，引导农民就近就地转移就业、返乡创业，增加劳务性收入；加大帮扶资金、直接补贴争取力度，增加转移性收入。继续抓好农民负担监管，防止反弹。

（二）加快转型升级，推进产业集聚壮大

优化升级增后劲。按照构建高质高效新产业的要求，加快实施“四大产业”发展计划，抓紧编制上下游产业链条布局方案，促进产业有效转移和要素合理配置，加快培育三大千亿产业集群。着力发展临港产业，加快大型钢铁项目前期工作，推动海西宁德工业区1000万方原油储备项目主体工程、华锐重工（宁德）大型机械设备制造等项目开工建设，促进联德镍合金、益联远大等项目尽早竣工投产。着力提升传统产业，推进电机电器、船舶修造、食品加工等传统产业创新转型和专业化分工。着力培育新兴产业，大力发展生物医药、新材料，支持柘荣海西药城、寿宁氧化锆、古田食品生物等生产基地加快建设。着力壮大海洋经济，重点培育海洋工程装备、游艇制造、海洋可再生能源、水产品深加工等海洋新兴产业，拓展提升滨海旅游、港口物流等海洋服务业，打造三都澳、白马港、福宁湾、沙埕港四大海洋经济聚集区。

激活存量促增长。突出抓调度优服务，指导企业用好用足支持实体经济发展的各类政策，争取出台省、市扶持船舶业健康发展的政策措施；加强168项工业重点新增长点的跟踪服务，加大小微企业帮扶力度，巩固经济增长的基本盘。突出抓技改促升级，完成技改投资80亿元以上。突出抓改制推上市，支持一批龙头骨干企业做大做强，力争新增上市企业2家以上。突出抓质量树品牌，新增中国驰名商标6件以上。

构筑平台强支撑。加强园区规划，建好产业集聚平台，创新建设投入机制，完善基础设施，提高园区自我发展能力和招商选资水平。发挥企业创新主体作用，用好“6·18”平台，密切产学研合作，支持龙头企业和重点科技型企业建立省级重点实验室、工程技术研究中心和企业技术中心，引导企业开展行业共性关键技术研究；落实人才高地政策，强化人才培养和引进，加强知识产权保护和运用。

壮大三产多贡献。出台扶持现代服务业发展政策措施，优化服务业结构，提升三产对经济增长的贡献率。加快发展旅游产业，争创太姥山5A级景区、白云山4A级景区，创新景区开发建设模式，提升景区交通和星级宾馆等配套设施，创建一批乡村星级接待点；完善旅游公共服务体系，推行旅游服务标准化试点；加快旅游线路整合，开拓国内外客源市场，打造以宁德世界地质公园为主体的旅游目的地。加快发展现代物流业，重点在产业集中区、港口道口、城市郊区打造一批物流园区和集散中心。推进城乡流通体系建设，建设和提升一批大型专业市场、农贸市场、商贸中心，继续实施“万村千乡市场工程”，加强重要商品、居民生活必需品的市场价格监测和调控保障。引导房地产市场健康平稳发展，增加普通商品住房有效供给。加快金融服务业发展，吸引更多金融机构入驻设点，引导金融网点向基层延伸，推动和扩大直接融资。培育发展服务外包、会展营销、电子商务等新型服务业态。

（三）强化项目带动，增强经济发展后劲

继续打好“五大战役”。拓展战役内涵，把海洋经济、旅游、生态等领域项目纳入战役范围，计划实施项目1290个，比去年增加127个，年度投资增长25%以上。强化战役保障，积极推进项目建设与土地开发、盘活资产以及引导社会资本投资一体化运作，全面推广和谐安征迁工作法，完善项目协调调度、跟踪服务和督查考评机制，确保项目建设顺利推进。促进战役接续，持续策划生成一批符合产业政策、带动力强的新项目；加快项目前期，落实项目业主责任制，建立财政性前期经费滚动使用管理机制，推动项目早开工早建设，实现梯次推进、滚动发展。

深化“三维”项目对接。围绕招大商引强企，办好第四届海峡两岸电博会暨第十四届宁德投洽会，提高各类招商活动市场化水平和目标客商参会率。进一步加强区域合作，积极实施“回归工程”，大力发展总部经济，更加注重产业链招商，提高“三维”对接项目的层次和质量。确保全年签约“三维”项目总投资超1000亿元，上年签约项目开工率80%以上，当年签约项目开工率50%以上。

（四）着眼宜业宜居，提升城乡建设水平

加快中心城市建设。启动滨海大道内侧及铁基湾围垦工程，科学有序推进中心城市临海布局、环湾发展。注重彰显闽东风格，高标准做好城市设计、建筑设计、景观设计，实施金溪流域、环东湖公园景观提升工程，加快一批城市综合体建设，提升中心城市形象和品位。统筹推进新区建设和旧城改造，加快建设福宁北路、闽东中路、富春东路等骨干路网，促进城市组团、新旧城区有效衔接；完善生活污水垃圾处理和配套设施，实施单石碑垃圾堆放场封场，加大城区内河湖泊整治力度，改造提升城市防洪排涝设施；健全城市管理体制机制，依法强力“清违”，持续整治环境卫生、交通秩序，加快发展城市公交，积极解决“停车难”问题，提高城市综合管理、精细管理水平，力争实现创建国家级园林城市和省级卫生城市目标。

拓展县域发展空间。不断增强福安、福鼎市区人口、产业集聚能力和综合承载功能，打造次级中心城市；积极实施“大城关”战略，提升县城规划、建设、管理水平，促进产业与城市空间布局协调互动，推进县城扩容提质，建设宜业宜居美丽县城。落实加快县域经济发展的政策措施，统筹规划县域产业发展，支持县（市）加快重点产业园区和集中区建设，因地制宜发展壮大特色县域经济。扶持山区县加快发展，鼓励山区和沿海共建产业园区，建立市县共建产业项目利益分配机制，推动形成各具特色、竞相发展、优势互补、互利共赢的县域发展格局。

促进镇村协调发展。坚持规划引领、产城并重，建设一批特色工业强镇、商业贸易重镇、历史文化名镇和现代农业大镇，加快城镇化进程，提高城镇化质量。实施好旧村复垦、增减挂钩、“三旧”改造，加快推进城镇公共设施建设，提升小城镇功能和形象。加快赛岐、太姥山、飞鸾、牙城等省级小城镇试点以及比照省级试点的南阳镇建设，提升14个市级小城镇试点建设发展水平。引导农村人口向城镇和中心村集聚，有序推进农业转移人口市民化。坚持镇村联动，加快推进新农村示范村建设，深入开展明星乡镇、明星村创建活动，每个县（市、区）突出抓好1个示范乡镇、2个示范村。全面推进“点线面”攻坚计划，打造一批宜居社区、宜居新村和交通主干道综合整治示范线路。实施“美丽乡村”建设行动，继续推动村庄规划编制，规范农村建房审批管理，加快推进以污水、垃圾处理为重点的农村环境整治，打造“天蓝、地绿、水净”的美好家园。

（五）完善基础设施，提高支撑发展能力

加快交通网络建设。按照构建畅通便捷新交通的要求，加快立体交通网络建设。支持公共码头、公共设施配套建设，建成白马作业区鼎信码头，加快建设城澳作业区沿线泊位和漳湾作业区8号、9号泊位，开工建设三都澳港区深水航道一期、白马作业区上饶码头、八尺门作业区通用泊位、城澳工作船基地和溪南作业区油储配套泊位等项目，提高港口泊位的吞吐能力。以“县县通高速”为目标，加快京台高速公路宁德段、福寿高速公路和沈海复线宁德至连江段、柘荣至福安段建设，力争开工建设沈海复线福鼎贯岭至柘荣段、福安至漳湾段和屏南至古田联络线、溪南疏港高速公路，加快推进福鼎闽浙界至太姥山段、城澳至古田高速公路前期工作。以“镇镇有干线”为方向，加快普通干线通乡镇公路提级改造，续建19条（段）、开工13条（段），建设农村公路283公里，提前一年完成农村公路安保工程。鼓励扶持农村客运发展，提高“村村通客车”水平。加快合福铁路宁德段、白马港铁路支线建设，全力推进衢宁铁路前期工作，力争尽快开工建设。

着力发展能源产业。加快宁德核电一期建设，力争2号机组年内投入运营。支持宁德新能源基地加快发展。推进宁德核电二期、大唐火电三期、周宁抽水蓄能电站、首钢控股天冠燃料乙醇、中海油宁德LNG等项目前期工作。开工建设古田溪一级电站改扩建工程、霞浦闾峡风电场。新建扩建各级变电站15座、110千伏及以上线路182公里。

（六）深化改革开放，激发发展活力动力

推进重点领域改革。加快农村综合改革步伐。稳步推进事业单位分类改革。稳妥有序推进税收“营改增”试点。建立全过程预算绩效评价机制和与财力相匹配的专项资金支出机制，创新政府性资金和投资项目监管机制。推动国有资产重组，促进市属国有骨干企业发展壮大，增强经营管理和投资运作的能力。

提升对外开放水平。积极争取三都澳、沙埕港水域扩大开放，加快口岸基础设施建设，促进通关便利化。落实鼓励外贸发展政策，推进外贸公共服务平台和省电机出口基地建设，对重点出口企业实行“一对一”服务，支持自主品牌和高附加值产品出口，鼓励企业建立境外营销平台和服务网络，巩固传统市场，开拓新兴市场，促进外贸出口持续稳定增长。有效扩大利用外资，加强外事侨务工作，密切友城关系，全方位、多层次开展对外交往。

深化宁台交流合作。推进宁台产业深度对接，加快建设台湾义联节能型合金综合利用园区。加强与台湾民间商会组织合作，深化对台经贸交流。加快推进霞浦台湾渔民创业园、屏南台湾生态农业园、寿宁闽台生态农业观光园、周宁茶博园建设，构建两岸山海资源合作平台。继续推进民间信俗和历史名人文化双向互动，促进宁台文化交流、旅游合作。

（七）建设文化强市，推动文化繁荣发展

加强核心价值体系建设。深入宣传贯彻党的十八大精神，积极开展社会主义核心价值体系、理想信念教育，大力弘扬民族精神、时代精神、福建精神和闽东精神。加强政务诚信、商务诚信、社会诚信和司法公信建设，注重人文关怀和心理疏导，培育自尊自信、理性平和、积极向上的社会心态。推进公民道德建设工程，提升市民文明素质，深化群众性精神文明创建活动，广泛开展志愿服务，营造知荣辱、讲正气、作奉献、促和谐的良好社会氛围。

实施文化惠民拓展工程。按照构建丰富多彩新文化的要求，加快一批文化事业项目的实施。建成市图书馆和妇女儿童活动中心，加快市大剧院、科技馆前期工作，推进公共文化资源免费开放，抓好乡镇综合文化站、社区文化活动中心、农家书屋的规范化、标准化建设，提高广播电视村村响、村村通质量，基本建成覆盖城乡的公共文化服务体系。做大做强“魅力闽东”文化惠民品牌，支持县（市）开展文化惠民演出，深入开展“三下乡”、“激情广场大家唱”、农村数字电影放映、文化艺术扶贫等活动，为人民群众提供更多高质量的文化服务。加强文化综合执法，净化文化市场。

传承发展闽东特色文化。丰富茶文化、畲族文化、红色文化、海洋文化、木拱桥文化、民间信俗文化内涵，推出一批彰显闽东文化特色的优秀文艺作品和广电栏目。做好文化遗产、历史文化名镇名村的保护和开发，加快闽东北木拱桥申报世界文化遗产进程，推进《宁德历史文化丛书》编纂和第二轮修志。

促进文化产业加快发展。全面落实文化产业扶持政策，继续推进文化产业“七个一”项目建设，加大文化产业招商力度，加快一批文化产业园区和项目建设。扶持发展畲族传统手工艺、木雕根雕、柘荣剪纸、蕉城仿古家具、霞浦树石盆景、古田双坑油画、寿宁乌金陶艺等特色文化产业，打造一批特色品牌。促进文化与旅游融合发展，培育新兴文化产业业态，丰富文化产业内涵。

（八）促进生态文明，着力打造美丽宁德

加强环境保护。突出责任落实，强化“一岗双责”，严格“一票否决”。突出节能减排，坚决淘汰落后产能，加快重点节能工程建设，推广使用节能产品；加强城乡污水处理厂建设和运营管理，完善雨污分流管网系统，推进工业园区污染集中处理，强化重点区域、重点行业、重点企业治污减排。突出流域治理，深化“两江三溪”重点流域水环境综合整治，开展饮用水源地专项整治。突出风险防范，加强重金属和危险废物环境管理，健全环境风险防范和应急管理体系。突出源头管控，按照“宁可少一点，也要环保一点”的要求，完善项目引进环境风险评估机制，强化对成长性企业的环保技术和政策指导。

推进生态创建。加快实施“四绿”工程，造林绿化39.7万亩。继续抓好水土保持生态修复、水土流失综合治理，加强生态和生物多样性保护。加强海洋环境监控，强力整治海上无序养殖，规范海上养殖秩序，坚决制止破坏海洋生态的各种行为。建立健全生态创建奖励补助和监督考核机制，创建国家级生态乡镇38个、省级生态乡镇26个、市级以上生态村274个，实现省级生态县全覆盖。

科学利用资源。落实基本农田管护措施，建设高标准基本农田4.5万亩，补充耕地1.45万亩，确保耕地占补平衡；加大土地收储力度，保障发展用地需求；依法清理闲置土地，节约集约利用资源。严厉打击违法采矿行为，严控一般采矿点审批，整合开发矿产资源。依法管好用好海洋资源，认真落实海域使用申请论证报批制度，逐步推行海域资源市场化配置，严格岸线、滩涂管制，严厉打击非法围填海，保护和有序开发无居民海岛。

（九）围绕幸福和谐，优先发展民生事业

健全社会保障体系。实施更加积极的就业政策，重点帮扶大中专毕业生、农业转移人口、零就业家庭、退役军人等群体多渠道多形式就业，鼓励青年创业。落实最低工资制度，健全职工工资正常增长和支付保障机制。加快保障性安居工程建设，新建各类保障房6013套、新增发放租赁住房补贴340户，完善配套管理机制。扩大各项社会保险覆盖面，逐步提高保障标准和水平，全面推进金保工程建设，基本建成市县乡村四级人力资源和社会保障服务平台。完善社会救助体系，抓好一批社会福利中心和养老机构建设与管理。更加注重帮扶弱势群体，提高城乡低保标准和革命“五老”、“五保户”供养水平，做好家庭困难学生资助工作，关注农村留守老人妇女儿童，做好关心下一代工作，重视发展社会福利、慈善、老龄、红十字和残疾人事业。

协调发展社会事业。加大各级财政投入力度，认真办好新一年36件市委、市政府确定的为民办实事项目。坚持教育优先发展。改扩建39所公办幼儿园，规范民办幼儿园发展，全面完成学前教育三年行动计划；建立校舍、校车安全保障长效机制，基本完成义务教育标准化学校建设，推进义务教育均衡发展；整合市县城区教育资源，扩大优质学校规模；提升高中教育质量，推行中职高职教育贯通培养模式，扩大紧缺技能型人才培养规模；启动宁德师范学院新校区三期工程建设，确保通过学士学位评估；关心特殊教育、老年教育、民族教育发展；加强教师队伍建设，逐步解决教师队伍结构性矛盾。着力提高人民健康水平，加强卫生人才队伍和基础设施建设，进一步扩充和优化配置医疗卫生资源，完善省市优质资源与基层卫生机构帮扶协作机制；抓好公立医院改革试点，强化县级医院能力建设，增加急需的薄弱学科床位，提高乡镇卫生院和村卫生所发展水平，重视基层中医药服务能力建设，不断提升医疗卫生服务质量；继续巩固和发展各类医保制度，推进医保支付方式改革。着力提高出生人口素质，促进人口长期均衡发展，实现省级计生优质服务县全覆盖。广泛开展全民健身运动，推进全民健身场地设施均等化建设，办好第四届市运会。认真做好老干部工作，继续做好人事、编制、统计、审计、档案、农机、气象、水文、防震减灾等工作。

大力创新社会管理。注重源头治理、动态管理、应急处理相结合，健全社会稳定风险评估机制和应急处置机制，严格按照“路线图”依法处理信访事项。推行社区管理网格化、信息化、精细化，加强社区矫正工作。支持工会、共青团、妇联等群团组织开展工作，引导社会组织健康有序发展。深入推进“六五”普法，积极开展法律援助，加强和改进律师工作。深化“平安宁德”建设，深入开展严打整治，推进“三位一体”治安防控体系建设。深化重点领域安全生产“打非治违”专项行动，继续开展道路交通安全综合整治“三年行动”。高度重视食品药品安全工作，加强基层食品药品安全体系建设，加大“餐桌污染”治理力度，建设食品放心工程。依法管理宗教事务。做好国防动员、人防、民兵预备役工作，争创新一轮省级双拥模范城。强化应急管理、海防、打私等工作。

三、努力建设廉洁高效、人民满意的服务型政府

认真贯彻落实中央关于改进工作作风、密切联系群众的八项规定和省委、市委的要求，不断加强和改进政府自身建设，提升服务发展、服务民生的能力和水平，以廉洁高效的服务对人民负责、让人民满意。

（一）以更加昂扬的精神推动发展。坚持与时俱进，不断解放思想，着力突破传统思维定势和运作方式，提高驾驭复杂局面和解决实际问题的能力。坚持弱鸟先飞，发扬滴水穿石精神，强化后发赶超意识，努力把潜在优势转化为现实优势和竞争优势。坚持力求先行，勇于担当、敢闯敢试，通过理念创新、方法创新、路径创新，破解难题、谋求跨越、促进发展。坚持规划引领，从更高的层面、更宽的视野谋划宁德新一轮的发展，科学编制“六新大宁德”建设的实施方案，扎实推进各项目标任务的落实。

（二）以更加务实的作风履行职责。牢记空谈误国、实干兴邦，常怀落实之心、常思落实之责、常谋落实之策、常抓落实之事、常验落实之效。把群众对美好生活的向往作为奋斗目标，深入开展群众路线教育实践活动，大力推行“一线工作法”，持续开展“四下基层、四解四促”活动，提高活动的实效；经常到困难和矛盾集中的地方问政问需问计，确保各项政策措施更加符合宁德实际、更加顺应群众期待。大力改进文风会风，不开没有实际意义的会议，不发照搬照抄、没有实质措施的文件，把更多时间和精力放在推动工作、解决问题上。

（三）以更加优质的服务营造环境。进一步深化行政审批制度改革，减少审批事项，精简审批流程，压缩审批时限。加强市县两级行政服务中心、乡镇便民服务中心、村（居）便民服务代办点建设，大力推行“马上就办”、容缺预审、并联会审等行之有效的做法，进一步提高审批效率。强化效能督查，严厉追究行政不作为，大力整治“庸懒散奢”、“推托顶慢”等不良风气，着力构建优质优美的新环境。

（四）以更加严明的纪律树立形象。依法接受人大及其常委会的工作监督和法律监督，主动接受政协民主监督，推进协商民主发展，提高人大代表建议、批评、意见和政协提案办理质量。强化政府层级监督和专门监督，主动接受社会公众监督和舆论监督，要求别人做到的市政府班子成员自己首先做到，要求别人不做的自己坚决不做。严格依法行政，规范权力运行，推进政务公开。厉行节约，严格控制一般性支出，把钱用在刀刃上。全面推进惩防体系建设，层层落实党风廉政建设责任制，加强反腐倡廉教育和廉政文化建设，严格执行领导干部重大事项报告制度，健全行政权力运行监督制约机制，始终保持惩治腐败力度，做到干部清正、政府清廉、政治清明。

各位代表，做好2013年工作，任务艰巨而光荣。让我们高举中国特色社会主义伟大旗帜，紧密团结在以习近平同志为总书记的党中央周围，在省委、省政府和市委的正确领导下，紧紧依靠全市人民，只争朝夕、真抓实干，不断夺取科学发展加快发展的新胜利，为构建“六新大宁德”，全面建成小康社会而努力奋斗！
